# When Is Rapid On-Site Evaluation Cost-Effective for Fine-Needle Aspiration Biopsy?

**DOI:** 10.1371/journal.pone.0135466

**Published:** 2015-08-28

**Authors:** Robert L. Schmidt, Brandon S. Walker, Michael B. Cohen

**Affiliations:** 1 Department of Pathology, University of Utah School of Medicine and ARUP Laboratories, Salt Lake City, Utah, United States of America; 2 ARUP Laboratories, Salt Lake City, Utah, United States of America; 3 Department of Pathology, University of Utah School of Medicine and ARUP Laboratories, Salt Lake City, Utah, United States of America; University of Toronto, CANADA

## Abstract

**Background:**

Rapid on-site evaluation (ROSE) can improve adequacy rates of fine-needle aspiration biopsy (FNAB) but increases operational costs. The performance of ROSE relative to fixed sampling depends on many factors. It is not clear when ROSE is less costly than sampling with a fixed number of needle passes. The objective of this study was to determine the conditions under which ROSE is less costly than fixed sampling.

**Methods:**

Cost comparison of sampling with and without ROSE using mathematical modeling. Models were based on a societal perspective and used a mechanistic, micro-costing approach. Sampling policies (ROSE, fixed) were compared using the difference in total expected costs per case. Scenarios were based on procedure complexity (palpation-guided or image-guided), adequacy rates (low, high) and sampling protocols (stopping criteria for ROSE and fixed sampling). One-way, probabilistic, and scenario-based sensitivity analysis was performed to determine which variables had the greatest influence on the cost difference.

**Results:**

ROSE is favored relative to fixed sampling under the following conditions: (1) the cytologist is accurate, (2) the total variable cost ($/hr) is low, (3) fixed costs ($/procedure) are high, (4) the setup time is long, (5) the time between needle passes for ROSE is low, (6) when the per-pass adequacy rate is low, and (7) ROSE stops after observing one adequate sample. The model is most sensitive to variation in the fixed cost, the per-pass adequacy rate, and the time per needle pass with ROSE.

**Conclusions:**

Mathematical modeling can be used to predict the difference in cost between sampling with and without ROSE.

## Introduction

Fine-needle aspiration biopsy (FNAB) is a widely used technique that is accurate and relatively free of complications. While palpation-guided FNABs are relatively inexpensive, image-guided and endoscopic techniques have increased the cost of FNAB sampling. In these settings there is a strong economic incentive to improve the reliability of sampling.[1] Rapid on-site evaluation (ROSE), or immediate cytologic assessment, can increase adequacy rates and reduce needle passes.[2–4] ROSE has the potential to reduce costs and reduce the number of needle passes because it can increase adequacy rates. In theory, ROSE should also reduce costs and morbidity associated with complications because it requires fewer needle passes. As a result, the demand for ROSE has increased, particularly for high-cost procedures such as Endobronchial ultrasound guided (EBUS) sampling and endoscopic ultrasound-guided (EUS) FNAB.

ROSE affects costs in several different ways. These effects act in opposite directions. ROSE increases adequacy rates that reduces the need for repeat procedures.[2, 4] This, in turn, reduces the costs as well as the morbidity and complications associated with repeat procedures. ROSE also decreases needle passes that, reduces morbidity.[5, 6] On the other hand, ROSE incurs additional costs because it requires additional personnel (cytologist) and increases procedure time. Thus, there is a trade-off between the cost savings and additional expenses.

Few cost-effectiveness studies exist on ROSE. Eedes, et al.[7] found that ROSE improved thyroid sampling performance but cost 220 minutes of cytologist time per additional diagnostic sample; however, this analysis did not account for the savings from fewer repeated samples. Eedes et al. concluded that ROSE should only be used for repeat FNABs. Nasuti, et al. calculated that ROSE would save approximately $356 per case. [8] Although the results of the Nasuti study (2002) were favorable, the study did not account for changes in procedure time resulting from ROSE. Layfield, et al.[9] found that the cost to perform ROSE is greater than the compensation received; however, this analysis only considered pathologist time and did not account for the impact of ROSE on the complete cost of care. Zanocco, et al. performed a cost-effectiveness analysis on ROSE for thyroid nodules.[10] Their model accounted for the competing effects of increased adequacy (fewer repeat procedures) and increased costs but did not account for the increase in procedure time. Several recent meta-analyses have shown that ROSE generally increases adequacy rates but these did not evaluate the cost-effectiveness of ROSE.[2–4, 11] Overall, the results on cost effectiveness of ROSE have been contradictory (Table 1). No studies have adequately accounted for the combined costs and benefits of ROSE.

**Table 1:** Summary of studies on the cost-effectiveness of rapid onsite evaluation (ROSE).

Study	FNAB Procedure	Setting	Results and Conclusion
Bruno, 2013[28]	Transbronchial needle aspiration	Italy	ROSE reduced costs by 19,400 euros over 60 cases (about $367 per case) by reducing the frequency of medianoscopy
Burgess, 2013[31]	Ultrasound guided,Head and neck,(thyroid, salivary glands, lymph nodes)	United Kingdom	ROSE would save approximately $164 per case if ROSE increased the adequacy rate to 100%.
Eedes, 2004[7]	Thyroid	USA, academic center	ROSE increased the diagnostic rate at a cost of 220 minutes per diagnostic sample. ROSE is only likely to be cost-effective in limited situations such as for repeat procedures.
Layfield, 2001[9]	Wide range of procedures: both image and palpation guided.	USA, academic center.	Cost of pathologist time to perform ROSE exceeds compensation by about $45 for image guided procedures. Compensation may be adequate only when samples are taken and immediately interpreted by the pathologist.
Nasuti, 2002[8]	5688 cases covering a wide range of anatomic sites	USA, academic center.	ROSE saved about $356 per case based on a total cost of $3096 per case.
Urquiza, 2007[32]	Endoscopic ultrasound guided FNA for gastro-intestinal lesions	Spain	Cost of sampling was $47 per correct diagnosis with ROSE and $49.5 per correct diagnosis without. ROSE was cost effective
Zanocco, 2013[10]	US guided thyroid	USA, academic center	The cost per quality adjusted life year was approximately $639,000 per case. ROSE is not cost effective.

The cost of ROSE depends on many factors. These factors include the skill of the aspirator, the accuracy of the cytologist, the number of needle passes, the length of the procedure, and the costs of personnel, equipment, and supplies. These factors will depend on the type of procedure and anatomic site and vary by location. Thus, the number of variables is large. Most clinical studies are limited because they only provide data on one or two variables at a time in a particular context. Thus, it would take a very large number of clinical studies to provide insight into the cost effectiveness of ROSE.

Mathematical modeling provides an alternative to clinical studies. Mathematical modeling is widely used to explain the relationships between sampling methods and costs.[12–15] Unlike clinical studies, mathematical models can explore a wide range of case scenarios and provide insight into the relationships between many variables. In these situations, mathematical modeling can provide insights that would be very difficult to obtain by clinical studies.

Two mathematical models have been developed that use simple sampling statistics to predict the impact of ROSE on FNAB sampling performance (adequacy rates, number of needle passes).[16, 17] In this study, we use these mathematical models to predict the impact of ROSE on costs. The overall objective of this study is to determine the conditions under which ROSE is cost effective.

## Methods

Mathematical models have been developed to predict the sampling performance of ROSE.[16, 17] Here, we build on those sampling models and develop a cost model to estimate the cost of sampling with and without ROSE. We then conduct computer experiments in which we use the model to determine under what conditions ROSE is cost effective.

### Overview

**Fig 1:**
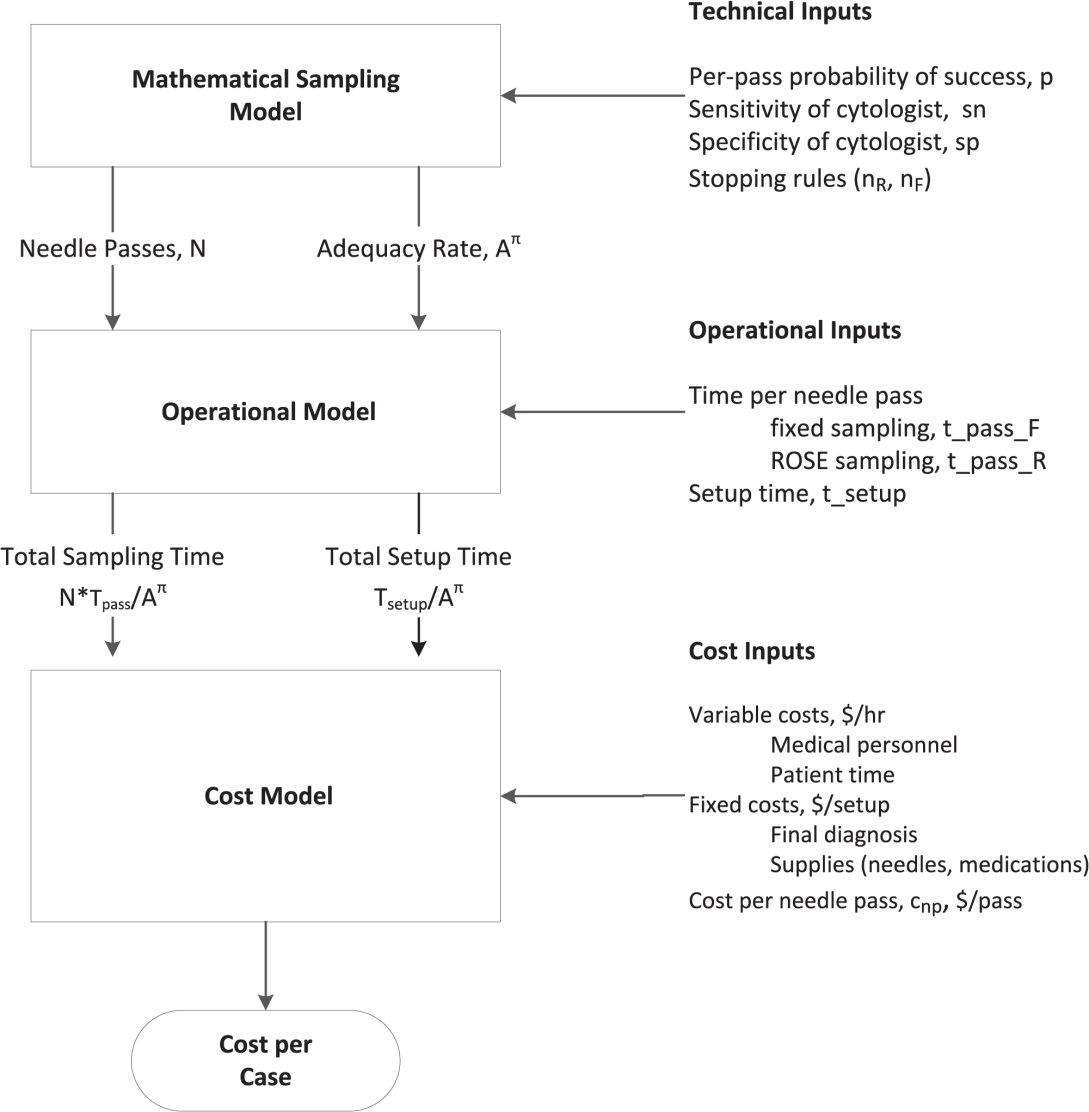
Sampling Algorithm. The case starts with a setup period. Setup costs depend on the length of time, personnel and other resources used during setup. The length of the sampling period depends on the sampling protocol. Each sampling protocol depends on a stopping condition. For fixed sampling, sampling stops after a fixed number of needle passes. In ROSE sampling, sampling stops after the cytologist observes a required number of adequate samples. The length of the sample period depends on the number of needle passes and the time per needle pass. Two kinds of costs are incurred during sampling: costs related to time (personnel costs) and costs related to needle passes (supplies, adverse events). After sampling, the samples are processed and evaluated. The case ends if the samples are adequate for diagnostic assessment. Otherwise, the procedure is repeated.

Our strategy is mechanistic (i.e., bottom-up). Our overall goal is to estimate the total cost per case. We did this in several steps. First, we estimated the total cost per procedure by estimating the variable and fixed costs for each *sampling protocol* (ROSE, no ROSE). (Italicized terms are defined in the Glossary, S2 Text). We estimated the variable cost by determining the time required to obtain an adequate sample and the resources consumed per hour. The total procedure time depends on the sampling method (time per needle pass and the expected number of needle passes). The total procedure time is composed of a set-up period and a sampling period. We estimated the total cost per procedure based on the duration for each period (setup, sampling) and the resource requirements ($/hr) for each period. We assumed that unsuccessful procedures (inadequate) are repeated. Thus, the total cost per case is given by the total cost per procedure multiplied by the expected number of procedures (see Fig 1). As shown below, the cost per procedure and the expected number of procedures depends on the sampling protocol. The model is described in detail in the Results Section under the heading “Theory: Model Development.”

### Computer Experiments

#### Scenarios

Our objective was to compare the performance of sampling with and without ROSE in common FNAB situations. We classified FNAB procedures into two generic *procedure types*: simple and complex. The simple procedure type was intended to represent FNAB procedures that would be performed in a physician’s office (e.g., palpation-guided FNAB or US-guided FNAB of thyroid) and require limited personnel and a short set-up time. The complex procedure represents procedures such as EUS-FNAB and CT-guided FNA that require more personnel and a longer setup time. The procedure type is designed to capture differences in the cost structure of different types of FNAB procedures. The scenarios are described in Table 2.

**Table 2:** Design of Computer Experiments. We investigated 32 scenarios which are described in the table below. Each scenario is a combination of a procedure type (simple or complex), a per-pass adequacy rate (low or high), and a set of sampling protocols (one for ROSE and one for fixed sampling). Procedure types are categorized as simple (palpation guided) or as complex (image guided). Simple procedures have shorter setup times and require fewer resources than complex procedures. Variable definitions are provided in Appendix (S1 Text). Sampling protocols are defined by stopping rules (number of needle passes for fixed sampling, and number of adequate samples for ROSE). Please see the text for additional explanation of procedure types, adequacy rates and sampling protocols. In the simple procedure: *t*_*setup*_ = 4 min, *c*_*var*,*o*_ = $30/hr, *c*_*var*,*c*_ = $100/hr, *c*_*np*_ = $0/pass and *c*_*pat*_ = $20/hr. In the complex procedure: *t*_*setup*_ = 30 min, *c*_*var*,*o*_ = $260/hr, *c*_*var*,*c*_ = $100/hr, *c*_*np*_ = $30/pass and *c*_*pat*_ = $20/hr.

Procedure Type	Per-Pass Adequacy Rate	Sampling Protocol		Scenario
		ROSE, *n*_*R*_	No ROSE, *n*_*F*_	
**Simple**	Low (*p* = 0.3)	1	1	2
			2	3
			3	4
			4	5
		2	5	2
			6	3
			7	4
			8	5
	High (*p* = 0.6)	1	9	2
			10	3
			11	4
			12	5
		2	13	2
			14	3
			15	4
			16	5
**Complex**	Low (*p* = 0.3)	1	17	2
			18	3
			19	4
			20	5
		2	21	2
			22	3
			23	4
			24	5
	High (*p* = 0.6)	1	25	2
			26	3
			27	4
			28	5
		2	29	2
			30	3
			31	4
			32	5

The adequacy rate represents the per-pass probability, *p*, of obtaining an adequate sample. The per-pass adequacy rate would vary on a wide range of factors such the lesion characteristics (size, solid vs cystic, anatomic location) and operator characteristics. The overall adequacy rate depends on the per-pass adequacy rate as well as the number of needle passes. Our model explicitly separates these two factors (per-pass adequacy, number of passes) to study the impact of different sampling protocols. We used two different per-pass adequacy rates (low vs high) to capture the impact of lesion and operator characteristics on sampling performance.

Sampling protocols are defined by a *stopping rule*. For sampling without ROSE, sampling stops after a fixed number of needle passes, *n*_*F*_. With ROSE, sampling stops after the cytologist observes a required number of adequate samples, *n*_*R*_. The relative performance of ROSE depends on the stopping rules. For that reason, we surveyed a wide combination of stopping rules. We compared sampling policies in which *n*_*R*_ varied from one to two adequate samples and *n*_*F*_ varied from two to five needle passes. This provided a total of eight sampling protocols which provided a comparison of particular combination of ROSE vs fixed sampling.

We defined a *scenario* as a comparison of ROSE vs fixed sampling with a specific combination of stopping rules (stopping rule for ROSE, stopping rule for fixed), per-pass adequacy rate (low vs high), and procedure type (simple vs complex). For example, a scenario might involve a comparison of ROSE (stopping after first adequate sample) and fixed sampling (three passes) in a simple procedure with a high per-pass adequacy rate. This created a total of 32 scenarios (4 fixed sampling stopping rules x 2 ROSE stopping rules x 2 per-pass adequacy rates x 2 procedure types). The scenarios are described in Table 2.

#### Sensitivity analysis

We conducted *one-way* and *probabilistic sensitivity analysis* (see Glossary, S2 Text) One way sensitivity analysis captures the impact of changing a single variable at a time. Probabilistic sensitivity analysis varies all variables and is able to capture the impact of interactions. For one-way analysis, we selected a base-case sampling protocol (*n*_*F*_ = 3, *n*_*R*_ = 1) and determined the impact of variation of *input parameters* (see Glossary, S2 Text) on the total cost. We performed one-way analysis on each of the four case-types. The input parameters for each case-type and the range over which each parameter was varied are presented in Table 3. One-way analysis was performed using @RISK (Palisade Corp, Ithaca, NY).

**Table 3:** Assumptions for Procedure Types. Procedure types were categorized as simple (palpation guided) or as complex (image guided). Simple procedures have shorter setup times and require fewer resources than complex procedures. Variable definitions are provided in Appendix (S1 Text).Each cell shows the base value and the range used for sensitivity analysis. Cells that span both columns indicate values that are the same for both types of procedures. Please refer to the Methods and Glossary (S2 Text) for a more detailed description of procedure types.

Parameter		Parameter Value—Mean (range)	
		Simple Procedure	Complex Procedure
Stopping point, ROSE	*n*_*R*_	1 adequate sample (1, 2)	
Stopping point, fixed	*n*_*F*_	3 needle passes (2–5)	
Sensitivity of cytologist	*sn*	0.95 (0.90–1.00)	
Specificity of cytologist	*sp*	0.975 (0.95–1.00)	
Per-pass adequacy rate	*p*	Low: 0.3 (0.2–0.4) High: 0.6 (0.5–0.7)	
Time per needle pass, fixed	$t_{pass}^{F}$	2 min (1–3)	
Time per needle pass, ROSE	$t_{pass}^{R}$	9 min (6–12)	
Setup time	*t*_*setup*_	4 min (3–5)	30 min (20–30)
Variable cost, other	*c*_*var*,*o*_	$30/ hr (20–40)	$260/ hr (190–330)
Variable cost, cytologist	*c*_*var*,*c*_	$100/hr (75–125)	$100/hr (75–125)
Total variable cost, fixed	$c_{var}^{F}$	$130/hr (20–40)	$260/hr (190–330)
Total variable cost, ROSE	$c_{var}^{R}$	$130/hr (95–165)	$360/hr (290–440)
Cost per needle pass	${\bar{c}}_{np}$	$0/pass	$30/pass (15–45)
Fixed cost per procedure	*c*_*fixed*_	$150(100–200)	$300 (150–450)
Cost to patient	*c*_*pat*_	$20/hr (15–25)	

We used probabilistic sensitivity analysis to study the overall variability in outcomes due to variation in all the inputs. We used Monte Carlo simulation to generate 100,000 samples for each of the 32 case *scenarios (*see Glossary, S2 Text). Each sample was obtained by randomly drawing input values from the ranges shown in Table 3. Using this method, we were able to determine the variation in outcome that would be obtained using a particular sampling protocol on a particular case-type. Probabilistic sensitivity analysis was performed using Stata (Stata Corp, College Station, TX).

### Theoretical Results: Model Development

#### Overview

**Fig 2:**
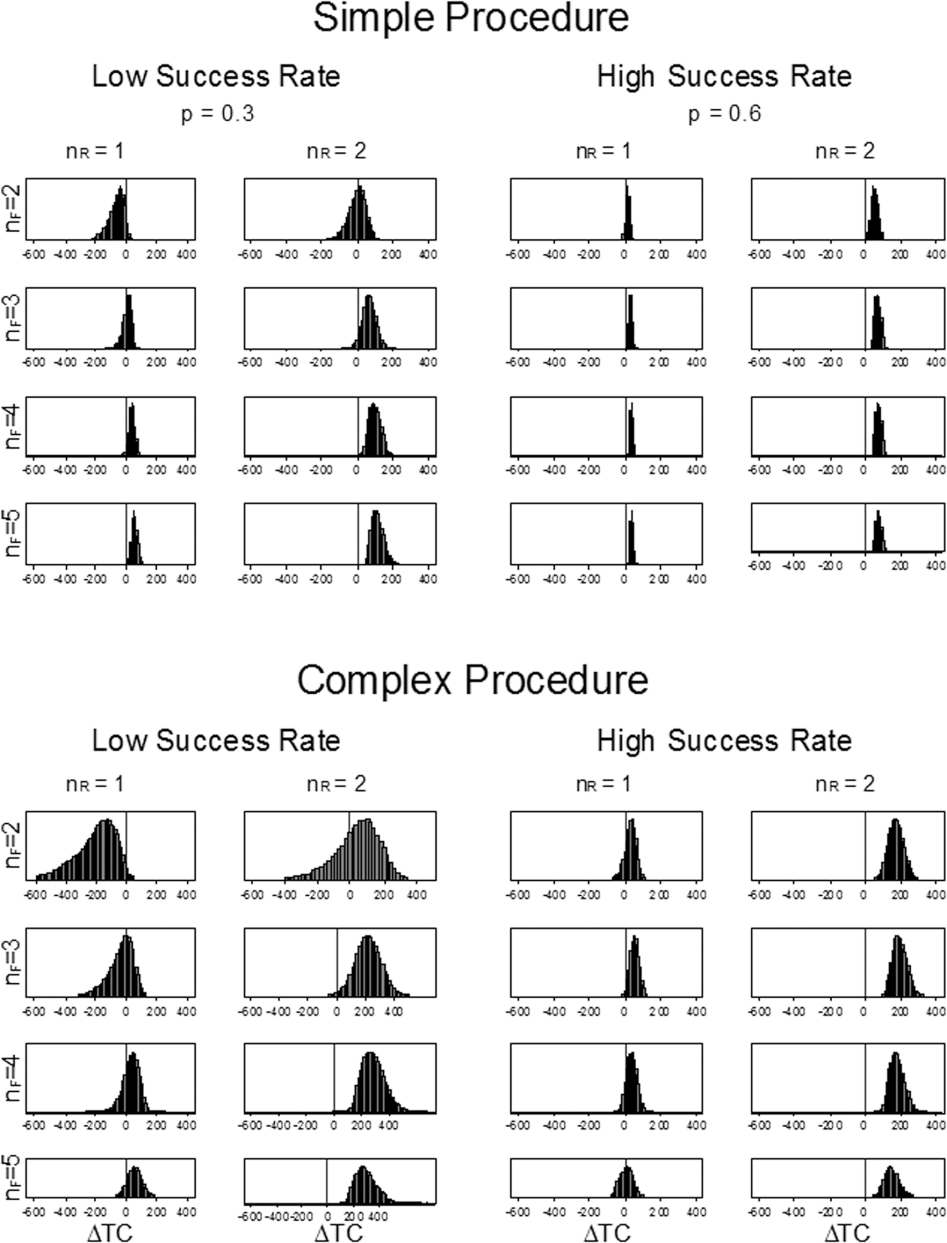
Explanation of cost analysis. The mathematical model estimates sampling performance (number of needle passes, number of procedures) based on a combination of controlled inputs (decision variables) and uncontrolled inputs (technical inputs). The operational model estimates operational performance (sampling time, setup time) based on the sampling performance and operational inputs. Finally, the cost model estimates the overall cost based on the operational performance and cost inputs.

Our model has three components: a sampling model, an operational model, and a cost model (Fig 2). The **sampling** model estimates the number of needle passes and the number of repeat procedures based on a set of technical inputs. The **operational** model estimates the total time per procedure. Finally, the **cost** model takes the outputs from the operational model and estimates the overall cost to obtain a diagnosis. Each component of the model is described in separate sections below.

#### Notation

Input variables (parameters) are indicated with lower case letters (e.g., *a*) and outcomes (*random variables*) are indicated with upper case letters (e.g., *A*). Expected values of outcome variables are indicated by a bar over the symbol (e.g., $\bar{A}$). Values for inputs and outcomes may be associated with a particular sampling protocol. We use a superscript to designate the sampling protocol: F for fixed sampling (i.e., non-ROSE) and R for ROSE (e.g., ${\bar{A}}^{F}$ or ${\bar{A}}^{R}$). We use the symbol, π, to indicate a generic sampling protocol (e.g., ${\bar{A}}^{\pi }$) Thus, ${\bar{A}}^{F}$ designates the expected value of outcome *A* with fixed sampling, ${\bar{A}}^{R}$ designates the expected value of outcome *A* with ROSE sampling, and ${\bar{A}}^{\pi }$ designates the expected value of outcome *A* for any sampling protocol. All variables are defined in the Appendix (S1 Text).

#### Sampling model: Number of Needle Passes and Adequacy Rate

The sampling model estimates the number of needle passes and the number of repeat procedures as a function of parameters that specify the sampling protocol. Overall, the sampling performance depends on five inputs: *sn*, *sp*, *p*, *n*_*F*_ *and n*_*R*_ (defined below). Collectively, we refer to these inputs as the technical inputs to the model. The technical inputs determine the sampling performance. Sampling performance is described in terms of two outcomes: the number of needle passes and the adequacy rate (via Eqs 2–6). These technical outcomes are used to estimate the operational performance of FNAB (Fig 2).

We assume a sampling process in which there is a constant per-pass probability, *p*, of success. A success is defined as an adequate sample (i.e., one with sufficient material for diagnostic assessment). The parameter, *p*, depends on many factors such as the experience and skill of the aspirator, the anatomic site, and the characteristic of the lesion (size, solid vs. cystic, etc). The overall sampling process consists of multiple needle passes and is considered successful if at least one needle pass produces an adequate sample. We use the term “per-procedure probability of success,” *P*(*S*), to designate the overall probability of success of multiple (as distinct from the per-pass probability, *p*). The per-pass probability, *p*, is a parameter of the model (i.e., an input) whereas the per-procedure probability of success, *P*(*S*), is a random outcome that depends on *p*. Importantly, the per-procedure success rate is equivalent to the adequacy rate.

In a fixed sampling protocol, sampling stops after a fixed number of needle passes, *n*_*F*_. Each needle pass can be viewed as a Bernoulli trial that has a probability, *p*, of producing an adequate sample. Let *X* be the total number of adequate samples obtained in *n*_*F*_ needle passes. In any procedure, the outcome, *X* can range from 0 to *n*_*F*_. The probability that *X* takes on some particular value, *k*, is given by the binomial distribution:

$$f\left(k|n_{F},p\right)=P\left(X=k\right)=\;\left(\begin{array}{c} n_{F}\\ k \end{array}\right)p^{k}\left(1-p^{k}\right)$$ (1)

A procedure is considered successful if at least if at least one sample is adequate. The adequacy rate is the probability, *P*(*S*), that a procedure is successful. Thus, the expected adequacy rate is given by

$${\bar{A}}^{F}=P\left(S\right)=\;P\left(X>0\right)=1-P\left(X=0\right)=1-{\left(1-p\right)}^{n_{F}}$$ (2)

where ${\bar{A}}^{F}$ is the expected adequacy rate of a fixed sampling protocol. The adequacy rate, ${\bar{A}}^{F},$ is the success rate of the overall procedure (result of multiple needle passes) whereas *p* represents the success rate of an individual needle pass.

We can verify the result in Eq 2. The procedure fails only if all *n*_*F*_ needle passes fail to produce an adequate sample. The probability that a single needle pass fails is (1 − *p*). Because each needle pass is independent, the probability that all *n*_*F*_ samples are inadequate is the product of the probability of failure of each individual needle pass, ${\left(1-p\right)}^{n_{F}}.$ The overall procedure has two possible outcomes: success or failure. Therefore, the probability of success is one minus the probability of failure: $1-{\left(1-p\right)}^{n_{F}}$.

In a fixed sampling protocol, there is no variation in the number of needle passes. The expected number of needle passes is:

$${\bar{N}}_{pass}^{F}=\;n_{F}$$ (3)

The binomial distribution is based on the assumption that the per-pass probability of success, *p*, is constant and is unaffected by the number of passes. This assumption is probably not strictly true in FNAB sampling because each needle pass disrupts the tissue and affects the probability of success of subsequent samples; however, the assumption that the per-pass probability of success is constant is a useful approximation and has been shown to be robust to deviations in which the probability of success declines with each needle pass.[18]

With ROSE, the number of needle passes varies. Each sample is assessed by a cytologist, and sampling stops when the cytologist observes a stopping point, *n*_*R*_. Most commonly, sampling stops after observing one adequate sample (i.e. *n*_*R*_ = 1) but the stopping point, *n*_*R*_, could vary depending on the type of case or the risk tolerance of the cytologist. We assume that *n*_*R*_ is set prior to sampling and could depend on physical characteristics of the lesion, clinical information, imaging studies, etc. In practice the stopping point, *n*_*R*_, might be adjusted during the procedure in light of the information obtained in prior samples. We regard *n*_*R*_ as the *average* number of adequate samples that a particular cytologist would require to cease sampling. For example, one cytologist may usually be able to render a decision after the first adequate sample (*n*_*R*_ = 1) whereas another cytologist may only feel comfortable after the second adequate sample (*n*_*R*_ = 1). Our model is flexible because it enables us to investigate the cost implications of these choices.

The accuracy of the cytologist is determined by the sensitivity (*sn*) and specificity (*sp*) of the adequacy assessment (*sn* and *sp* of the cytologist are judged relative to the final cytological assessment after processing). We assume that the sensitivity and specificity are known or estimated. In general, the sensitivity and specificity of the cytologist, *sn* and *sp*, could depend on a number of factors such as experience level, the anatomic site, etc. Under these circumstances, the overall probability of success depends on the per-pass probability of success as well as the accuracy (*sn* and *sp*) of the cytologist. For example, a false positive assessment would terminate sampling and could lead to sampling failure. False negative assessments would result in unnecessary needle passes. The expected adequacy rate and expected number of needle passes for variable sampling (ROSE) with assessment by an imperfect cytologist are given by:[16]

$${\bar{A}}^{R}=P(S)=1-P(failure)==1-{\left[\frac{\left(1-sp)(1-p\right)}{1-sp(1-p)}\right]}^{n_{R}}$$ (4)

$${\bar{N}}_{pass}^{R}=\;\frac{n_{R}}{(1-sp)(1-p)+p*sn}$$ (5)

We assume that the FNAB procedure is repeated if it fails. The expected number of required procedures is:

$${\bar{N}}_{proc}^{\pi }=\;\frac{1}{{\bar{A}}^{\pi }}$$ (6)

where π indicates the type of sampling protocol (fixed or ROSE). We provide evidence that our model can predict clinical outcomes in the Supporting Material (S4 Text).

#### Operational Model: Time per procedure

The operational model estimates the total time required for each procedure using estimates of the number of needle passes obtained from the sampling model.

Our general strategy is to divide the procedure into different time periods and to estimate the resource consumption in each time period. Resource use may vary during the procedure and, in principle, one could divide the procedure into many different time periods to account for pattern of resource use.[19] Thus, the cost for each period would be the length of the period times the sum of the hourly cost of the resources used in each period, *t*_*i*_ ∑_*j*_ *cij*. The total variable (i.e. time-based) cost would be the sum of the costs in all periods: ∑_*i*_(*t*_*i*_ ∑_*j*_ *cij*).

For simplicity, we divided the procedure time into two periods: setup and sampling. Let *t*_*setup*_ be the time required for set up. Let $t_{pass}^{\pi }$ be the time required per needle pass under sampling protocol, π. (The symbol, π, is used to designate *any* sampling protocol. We use the symbols F and R to designate a specific sampling protocol). The time per needle pass depends on the sampling protocol. In general, the time per needle pass is longer for ROSE sampling. For example, the time per pass for fixed sampling, $t_{pass}^{F},$ might be 3 minutes and the time per pass for ROSE sampling, $t_{pass}^{R}$ 4, might be 8 minutes. We refer to the time per needle pass and setup time as operational inputs (Fig 2). The total sampling time is given by the time per needle pass times the expected number of needle passes:

$${\bar{T}}_{samp}^{\pi }=\;{\bar{N}}_{pass}^{\pi }\;t_{pass}^{\pi }$$ (7)

Eq 7 shows that the sampling protocol has two effects on the total sampling time. First, it affects the expected number of needle passes, ${\bar{N}}_{pass}^{\pi }.$ Second, the time per needle pass, $t_{pass}^{\pi },$ also depends on the sampling protocol. ROSE tends to decrease the number of needle passes but increases the time per needle pass. The total procedure time would be the sum of the setup time and the sampling time:

$${\bar{T}}_{proc}^{\pi }=\;t_{setup}+{\bar{T}}_{samp}^{\pi }\;=\;t_{setup}\;+\;{\bar{N}}_{pass}^{\pi }\;t_{pass}^{\pi }$$ (8)

We assume that the setup time is independent of the sampling protocol ($t_{setup}=t_{setup}^{F}=t_{setup}^{R}).$ For example, the setup time for an EUS-FNAB does not depend on whether or not ROSE is used. In our model, the variation in total procedure time is driven by the variation in sampling time which, in turn, is determined by the expected number of needle passes, ${\bar{N}}_{pass}^{\pi },$ and the time per pass, $t_{pass}^{\pi }.$ Both of these quantities depend on the sampling protocol as indicated by the superscript, π.

#### Cost Model

We use a *societal perspective* as recommended by current guidelines.[20, 21] The societal view includes all medical costs, costs to the patient, and costs to society such as lost productivity. The societal perspective is preferred because it incorporates more limited perspectives such as the government, medical, or payer perspectives. It is easy to modify a model based on the societal perspective to create a model based on more limited perspective (e.g. government, medical or payer perspectives).

We categorized costs as time-dependent (variable) and procedure-dependent (fixed). Time-dependent costs include personnel as well as items such as equipment and space that incur an *opportunity cost* as a function of time. (The opportunity cost is the cost of the forgone alternative. For example, the opportunity cost of endoscopy suite time is the profit that would be obtained if that time could be used to schedule an additional procedure). For convenience, we distinguish variable costs that are incurred by the cytologist from all other variable costs (the cost of the cytologist is the major difference between the hourly cost of ROSE and fixed sampling). The overall hourly variable cost for each policy is:

$$c_{var}^{F}=\;c_{var,c}+c_{pat}$$ (9A)

$$c_{var}^{R}=\;c_{var,c}+\;c_{var,o}+\;c_{pat}$$ (9B)

where

*c*_*var*,*c*_ = Variable cost of the cytologist, $/hr.

*c*_*var*,*o*_ = All other variable costs aside those associated with cytologists, $/hr.

*c*_*pat*_ = Hourly wage of the patient (only included in societal perspective)

The cost of the cytologist would vary depending on whether the procedure is performed by an on-site cytopathologist, on-site cytotechnologist, or a combination of telcytology cytopathologist and an anciallary staff person. Our model can account for all these contingencies by adjusting the variable cost parameters, *c*_*var*,*c*_ and *c*_*var*,*o*_.

Variable costs are incurred in two different time periods: setup and sampling. For simplicity, we will assume that the variable costs are approximately the same during setup and sampling periods. We also assume that $c_{var}^{other}$ is the same for both fixed and ROSE sampling. Thus, the total variable costs of fixed and ROSE policies differ by the cost of the cytologist. The expected total variable cost is the product of the variable cost and the time required for each period.

$${\bar{TC}}_{var}^{\pi }=\left(t_{setup}\;+{\bar{T}}_{samp}^{\pi }\right)c_{var}^{\pi }$$ (10)

Fixed costs are defined as costs that are independent of time and are incurred as a function of a procedure. Most commonly, the fixed costs would be supplies. For simplicity, we assume that the fixed costs, *c*_*fixed*_, are independent of the sampling protocol ($c_{fixed}=c_{fixed}^{F}=c_{fixed}^{R}$).

Our model uses the number of needle passes to estimate the time of the sampling period. Each needle pass not only consumes time but is also associated with morbidity. Let ${\bar{c}}_{np}$ be the *expected cost* of a needle pass. This quantity includes the weighted average of the probability of each type of adverse event and its associated cost.

The expected total cost per-procedure associated with a particular sampling protocol is given by the sum of the variable (time dependent), fixed (procedure dependent) and the costs associated with needle passes.

$${\bar{TC}}_{proc}^{\pi }=\;{\bar{TC}}_{var}^{\pi }+c_{fixed}=\;\left(T_{setup}\;+{\bar{T}}_{samp}^{\pi }\right)c_{var}^{\pi }+\;c_{fixed}+\;{\bar{N}}_{pass}^{\pi }{\bar{c}}_{np}$$ (11)

We assume that a procedure is repeated if it fails to obtain at least one adequate sample. The total overall *expected* cost per case is given by the cost per procedure times the expected number of procedures:

$${\bar{TC}}^{\pi }=\;{\bar{TC}}_{proc}^{\pi }{\bar{N}}_{proc}^{\pi }\;=\;\frac{\left(t_{setup}\;+{\bar{T}}_{samp}^{\pi }\right)c_{var}^{\pi }+\;c_{fixed}+\;{\bar{N}}_{pass}^{\pi }{\bar{c}}_{np}}{A^{\pi }}$$ (12)

After substituting equations, we obtain the following estimates for the expected cost of FNAB sampling with and without ROSE. The expected cost of fixed sampling (i.e. without ROSE) is given by:

$${\bar{TC}}^{F}\;=\;\frac{\left(t_{setup}+\;t_{pass}^{F}n_{F}\right)\;\left(c_{var,o}+c_{pat}\right)+\;c_{fixed}+\;{\bar{N}}_{pass}^{F}{\bar{c}}_{np}}{1-{\left(1-p\right)}^{n_{F}}}$$ (13A)

The expected cost of sampling with ROSE is given by:

$${\bar{TC}}^{R}\;=\;=\frac{\left(t_{setup}+(t_{pass}^{R}+\frac{{\bar{c}}_{np}}{c_{var}^{R}}\left(\frac{n_{R}}{\left(1-sp\right)\left(1-p\right)+p*sn}\right)\right)c_{var}^{R}+c_{fixed}}{\left(1-{\left[\frac{\left(\left(1-sp\right)\left(1-p\right)\right)}{\left(1-sp\left(1-p\right)\right)}\right]}^{n_{R}}\right)}$$ (13B)

where $c_{var}^{R}$ = $c_{var,c}+\;c_{var,o}+\;c_{pat}$.

The overall difference in the expected cost per case is:

$$\bar{\Delta TC}=\;TC^{R}-TC^{F}$$ (14)

Eqs 13A and 13B show how the cost per diagnosis can be estimated from decision variables **(**type of sampling: fixed or ROSE, the number of required samples or stopping point, *n*_*F*_ or *n*_*R*_), technical parameters (*p*, *sn*, *sp*), operational parameters ($t_{setup},\;t_{pass}^{F},\;t_{pass}^{R}$), and cost parameters $\left(c_{var}^{cyto},c_{var}^{other},\;c_{fixed},c_{np}\right)$ as shown in Fig 2. Our model (Eq 13) is based on several simplifying assumptions. We provide a general model in the Supporting Material (S3 Text).

#### Input Assumptions

As noted in the overview, our model depends on three types of inputs: **technical** inputs, **operational** inputs, and **cost** inputs. We assumed values for each of these inputs. The assumptions are listed in Table 2 and are described below.

#### Technical inputs

The technical inputs include the sampling protocol (*n*_*F*_, *n*_*R*_), the per-pass success rate (*p*), and the accuracy of the cytologist (*sn*, *sp*). We assumed that *n*_*F*_ varied between two and five passes and *n*_*R*_ varied between one and two adequate samples. For the base-case sampling protocol (used in one-way analysis), we assumed that fixed sampling uses three passes (*n*_*F*_ = 3) and that ROSE sampling stops after observing one adequate sample (*n*_*R*_ = 1). We assumed that the cytologist has a sensitivity of 0.95 and a sensitivity of 0.99 for sample accuracy.[22–26] Previous studies have shown that the benefits of ROSE depend on the per-pass probability of success. We compared two different levels of sampling success: low per-pass success rate (*p* = 0.3) and high per-pass success rate (*p* = 0.6). With a fixed sampling policy using three passes, the per-case probability of success would be 66% and 94%, respectively for low and high per-pass probability of success. These per-case probabilities of success represent the low and high values typically reported in adequacy studies.[11]

#### Operational inputs

We assumed that the setup time was three minutes for simple procedures and 20 minutes for complex procedures (please refer to the section on Scenarios for definition of simple and complex procedures). The time per needle pass depends on the sampling protocol. [9, 27] We assumed that the time per needle pass was 2 minutes for fixed sampling and 9 minutes for ROSE sampling.

#### Cost inputs

We used a *micro-costing* approach to estimate costs. We assumed that all procedures incurred a fixed cost of $150 for final cytopathological diagnosis. [10] We assumed that complex procedures require additional supplies (e.g., anesthesia, special needles) that add $150 to the fixed costs. So, the total fixed cost, *c*_*fixed*_, was $150 for simple procedures and $300 for complex procedures. We assumed that simple procedures required a technician and either a physician or cytologist. We assumed technician time cost $30 per hour and physician/cytologist time cost $100 per hour. Thus, the variable cost for a simple procedure was $130 per hour. We assumed that complex procedures required a specialist (radiologist or endoscopist) and two assistants (technicians, nurses) at $30 per hour. We assumed specialist time cost $200 per hour. Thus, the baseline variable cost (*c*_*var*,*o*_) for complex procedures was $260 per hour. With ROSE sampling, the cost of the cytologist added an additional $100 per hour so the total variable cost for a complex procedure with ROSE was $310 per hour. We assumed the cost per needle pass, *c*_*np*_, was zero for simple procedures and $30 per pass for complex procedures (accounting for morbidity, special needles, etc).

## Experimental Results

As shown in Eqs 13 and 14, the expected cost difference between ROSE and fixed sampling, $\bar{\Delta TC},$ depends on 11 parameters: ${\bar{c}}_{np},c_{fixed},c_{pat},c_{var,c},c_{var,o},n_{F},n_{R},p,t_{pass}^{F},,t_{pass}^{R},t_{setup}$.

The precise impact of each parameter depends on the value of the other parameters; however, several trends are apparent. We conducted a one-way sensitivity analysis by varying the value of each parameter in Eqs 13A and 13B. ROSE is favored when (group by parameter type):

### Technical factors

- the per-pass probability of success, *p*, is low
- the number of passes, *n*_*F*_, in fixed sampling is low
- the accuracy of the cytologist, *sp* and *sn*, is high.

### Operational factors

- the setup time, *t*_*setup*_, is long
- the difference between the times per pass, $t_{pass}^{R}-$ $t_{pass}^{F},$ is low

### Cost factors

- the fixed costs, *c*_*fixed*_, are high
- the total variable cost, $c_{var}^{\pi }=c_{var,o}+\;c_{var,c}+\;c_{pat}$, is low
- The cost per needle pass, *c*_*np*_, is high
- ROSE stops after observing one adequate sample (*n*_*R*_ = 1)

We compared fixed and ROSE sampling in four different case scenarios using the base-case sampling protocol (*n*_*F*_ = 3, *n*_*R*_ = 1). We found that ROSE was most advantageous when the per-pass success rate was low (Table 4). On average, sampling with ROSE saved $37 per case in complex procedures when the per-pass probability of success was low. When the per-pass probability of success was high, ROSE cost $53 more than fixed sampling in complex procedures. ROSE was more costly when used in simple procedures, irrespective of the per-pass probability of success.

**Table 4:** Comparison of ROSE vs fixed sampling for four case types using the baseline sampling protocol. (Stopping rules: 3 needle passes for fixed sampling, 1 adequate sample for ROSE). Case types correspond to the procedure type (simple vs complex) and the per-pass probability of an adequate sample (low vs high).

Outcome		Symbol	Scenario			
			Complex High Success	Complex Low Success	Simple High Success	Simple Low Success
**Expected Total Cost**	**Rose**	***TC*** ^***R***^	$652	$836	$203	$251
	**Fixed**	***TC*** ^***F***^	$599	$874	$170	$248
	**Difference**	**difference**	$53	-$37	$33	$3
**Needle Passes per Procedure**	**Rose**	$N_{\mathrm{pass}}^{R}$	1.7	3.4	1.7	3.4
	**Fixed**	$N_{\mathrm{pass}}^{F}$	3.0	3.0	3.0	3.0
	**Difference**	**difference**	-1.3	0.4	-1.3	0.4
**Adequacy Rate**	**Rose**	***A*** ^***R***^	0.98	0.94	0.98	0.94
	**Fixed**	***A*** ^***F***^	0.93	0.65	0.93	0.65
	**Difference**	**difference**	0.05	0.29	0.05	0.29
**Number of Procedures Per Diagnosis**	**Rose**	$N_{\mathrm{proc}}^{R}$	1.02	1.06	1.02	1.06
	**Fixed**	$N_{\mathrm{proc}}^{F}$	1.07	1.57	1.07	1.57
	**Difference**	**difference**	-0.05	-0.51	-0.05	-0.51
**Cost Per Procedure**	**Rose**	${\mathrm{TC}}_{\mathrm{proc}}^{R}$	$641	$787	$199	$237
	**Fixed**	${\mathrm{TC}}_{\mathrm{proc}}^{F}$	$558	$558	$158	$158
	**Difference**	**difference**	$83	$229	$41	$79
**Duration Of Sample Period**	**Rose**	$T_{\mathrm{sample}}^{R}$ **(min)**	15.6	30.6	15.6	30.6
	**Fixed**	$T_{\mathrm{sample}}^{F}$ **(min)**	6.0	6.0	6.0	6.0
	**Difference**	**difference**	9.6	24.6	9.6	24.6

**Fig 3:**
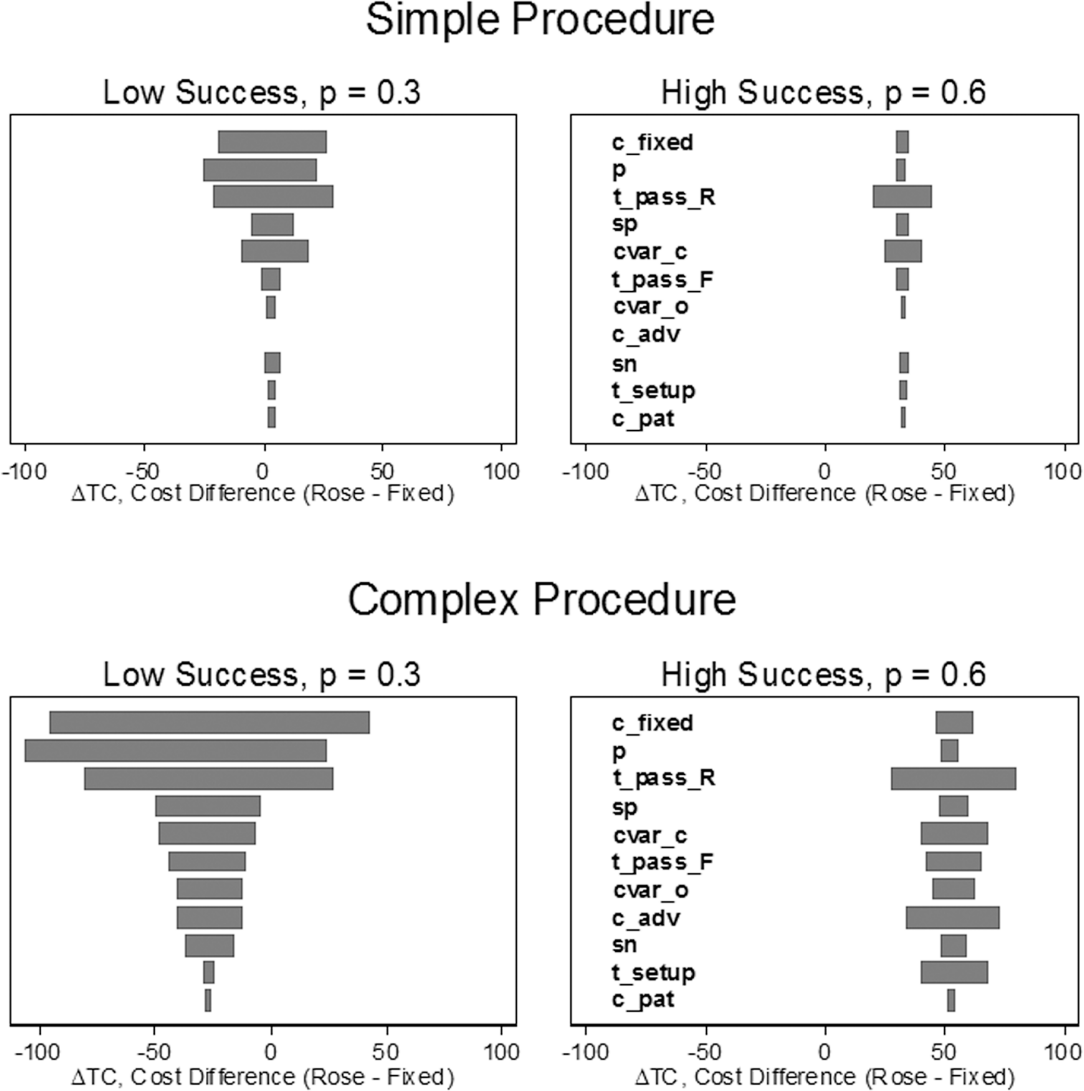
Probabilistic sensitivity analysis of cost difference of ROSE and fixed sampling by scenario. The graph shows the distribution of the difference in expected cost (ROSE–fixed) for 32 different scenarios. Scenarios consist of a procedure type (simple or complex), a per-pass success rate (low or high) and stopping rules for sampling (fixed sampling stops after *n*_*F*_ needle passes, ROSE stops after *n*_*R*_ adequate samples are obtained). For each scenario, the input parameters were varied over the range shown in Table 2 using Monte Carlo simulation. Values less than zero favor ROSE. For example, ROSE is generally less costly than fixed sampling in the scenario in the upper left panel (simple procedure, low per-pass success rate, *n*_*R*_ = 1, *n*_*F*_ = 2).

We conducted a more detailed analysis in which we conducted a probabilistic sensitivity analysis for all 32 combinations of case- and sampling protocols (Fig 3). In general, ROSE was more costly than fixed sampling when the per-pass success rate was high or when the fixed sampling protocol used a high number of needle passes (*n*_*F*_ = 5). ROSE was less costly only when the per-pass success rate was low. The fixed sampling protocol used two passes (*n*_*F*_ = 2), and ROSE sampling stopped after the first adequate sample (*n*_*R*_ = 1). There were two conditions were there was no significant cost difference: 1) when the fixed sampling protocol used three needle passes, or 2) if ROSE stopped after two samples (*n*_*R*_ = 2) and fixed sampling stopped after two needle passes. In general, the variance in the cost difference was smaller for simple procedures than for complex procedures and when the per-pass success rate was high (Fig 3).

The total cost per procedure, ${\bar{TC}}_{proc}^{\pi },$ was greater for ROSE than for fixed sampling (Table 4). The increased cost per procedure was due to the increase in sample time, ${\bar{T}}_{samp}^{\pi }$, associated with ROSE. ${\bar{T}}_{samp}^{R}$ was 9.6 minutes longer than ${\bar{T}}_{samp}^{F}$ when the per-pass success rate was high and was 24.6 minutes longer when the per-pass success rate was low. The cost-increasing effects were counter-balanced by the impact of ROSE on repeat procedures, ${\bar{N}}_{proc}^{\pi }$. The number of repeat procedures with ROSE was approximately 60% of the repeat procedures with fixed sampling (${\bar{N}}_{proc}^{R}=1.06,\;{\bar{N}}_{proc}^{F}=1.57)$ when the per-pass probability of success was low.

**Fig 4:**
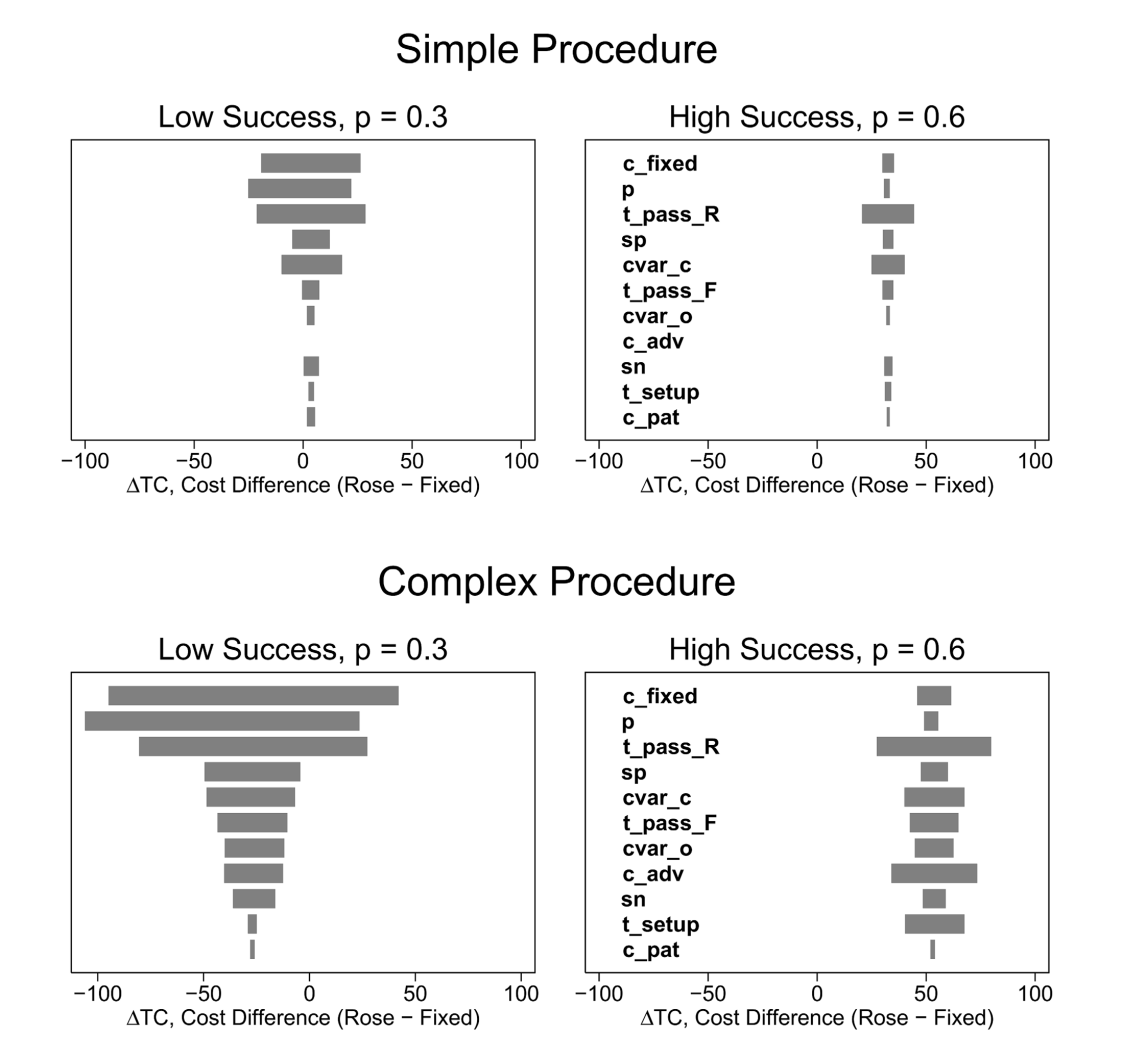
One-way sensitivity analysis. Each input parameter was varied over its range (shown in Table 2) and the difference in total cost per case (ROSE–fixed) was calculated for each value of the input parameter. The total cost difference is given by Eq 14 in the text. Negative values indicate that ROSE is less costly. Each bar shows the range of the difference in total cost as a function of variation in a particular variable. For example, the top bar in each panel shows the sensitivity of the difference in total cost to variation in the fixed cost. Each panel shows the one-way sensitivity of a particular scenario to variation in the input parameters. Wide bars indicate the cost difference is relatively sensitive to variation in the parameter. Narrow bars indicate low sensitivity. Each scenario consists of a procedure type (simple or complex), a per-pass success rate (low or high) and a set of stopping rules. Each scenario used the same set of stopping rules (three needle passes for fixed sampling, ROSE stops after observing the first adequate sample). Please refer to the Glossary (S2 Text) or the text for definitions of procedure types, and scenarios. Variables are defined in Appendix (S1 Text) and in the text.

One-way sensitivity analysis showed that difference in total cost per diagnosis was most sensitive to the fixed cost, *c*_*fixed*_, the per-pass probability of success, *p*, and the time per needle pass for ROSE sampling, $t_{pass}^{R}$ (Fig 4). The total cost difference (ROSE–fixed) was positive over the entire plausible range of each input variable when the per-pass success rate, *p*, was high. The cost difference was most sensitive to the input parameters when the per-pass probability of success was low and when the procedure type was complex.

## Discussion

We identified 10 parameters that affect the overall cost of sampling and developed a mechanistic model that shows how these factors affect the cost of sampling. We divided these into technical, operational, and cost parameters. We were able to identify general case scenarios in which ROSE is less costly than fixed sampling policies. Overall, our model captures the tradeoff between the extra time and expense required for ROSE and the reduction in repeat procedures.

Further, our model explains findings that have been observed in clinical studies. For example, clinical studies have shown that ROSE improves the adequacy rate only when the probability of success is low. Our model explains this in terms of a binomial sampling process that depends on the number of needle passes and the per-pass success rate of each pass. In ROSE sampling, the number of needle passes depends on the specificity and the stopping rule (number of adequate samples required to stop sampling). The stopping rule, *n*_*R*_, can be viewed as an indicator of confidence. A confident cytologist might be willing to stop sampling after observing a single adequate ample (*n*_*R*_ = 1) where a less confident cytologist might wish to observe two adequate samples before stopping. Our analysis shows (Fig 3) that the advantages of ROSE are negated when additional samples are required.

In general, the cost differences between ROSE and fixed sampling were small (less than $50 per case) when we used base case stopping rules (*n*_*F*_ = 3, *n*_*R*_ = 1) (see Table 4). We may have observed smaller differences because we assumed that the procedure was repeated. Bruno, et al. assumed patients would be referred for medianoscopy following a failed transbronchial FNAB (TBNA) procedure and attributed a savings of $267 per case to ROSE due to the reduced rate of medianoscopies.[28] ROSE reduces the inadequacy rate and therefore provides greater savings if failed procedures are followed by more expensive procedures such as medianoscopy. Our assumption (FNAB was repeated following an inadequate procedure) was conservative and the estimated savings for complex procedures ($37) may represent a lower bound.

ROSE can reduce the costs associated with repeat procedures. Repeat procedures incur unnecessary costs associated with repeat setups. Thus, ROSE is most advantageous when the setup costs are relatively high. This occurs in complex procedures because the setup time is longer and the cost per hour is higher than in simple procedures. Repeat procedures are also more likely when the per-case adequacy rate is low. For a fixed sampling protocol, the per-case adequacy rate increases with the per-case adequacy rate and the number of needle passes. Thus, ROSE is advantageous when compared to fixed sampling policies using a low number of needle passes and when the per-pass adequacy rate is low. Overall, our results suggest that ROSE only provides a cost advantage when compared against fixed sampling policies with relatively low adequacy rates in procedures with relatively high setup costs (i.e. complex procedures).

ROSE also reduces needle passes which reduces the morbidity and costs. Thus, ROSE would be favored in situations with relatively high rates of adverse events. For example, CT-guided FNAB of the lung can cause pneumothorax which can require insertion of a chest tube and hospital admission. Our model accounts for adverse events by including a cost per needle pass. ROSE would be favored in circumstances where the cost per needle pass is relatively high.

Our model rests on several assumptions. First, it relies on a simple binomial sampling model that may not provide an accurate representation of actual FNAB sampling. In particular, the binomial sampling model assumes that the per-pass probability of success is constant and does not vary with the number of needle passes. This assumption is probably not true because each needle pass affects the target so that the probability of success decreases with the number of needle passes. While this scenario seems plausible, no studies have shown that the per-pass probability of success depends on the number of needle passes. On the other hand, we are aware of two studies that show that the binomial sampling model provides a very good fit for FNAB sampling.[29, 30] In addition, predictions of the binomial model have been shown to be relatively robust to deviations from the constant-probability assumption.[18] Second, our model assumes idealized behavior in which the cytologist picks the stopping rules (*n*_*R*_, *n*_*F*_) prior to the procedure and never deviates from this rule. This assumption was necessary because we wanted to compare the performance of specific variants of ROSE and fixed sampling (e.g. fixed sampling with *n*_*F*_ = 3 against ROSE with *n*_*R*_ = 1. In practice, sampling is less rigid and the stopping rule is sometimes determined by information gained from prior samples. For example, one might initially plan to use three needle passes in a fixed sampling policy but then decide to take additional passes On the other hand, our model is flexible and could be extended to predict the impact of deviations from idealized policies that would be likely to occur in practice. Finally, our model assumes that the costs can be adequately modeled by dividing the total time into a setup time and a sampling time, and by determining the cost (resource use) per hour. We made the simplifying assumption that the variable cost was the same for both periods (setup and sampling); however, our model is flexible and could be modified to account for resources that varied by period (setup vs sampling). Additional periods (e.g., cleanup, stepdown) could also be added. Costs and procedures vary by institution so it is impossible to cover every possible variation. Our objective was to illustrate a flexible approach that can be easily adapted to provide estimates in a wide range of settings.

Our calculations were also based on many assumptions for the input parameters. We conducted extensive sensitivity analyses (one-way, probabilistic, and scenario based) to determine the impact of uncertainty in the inputs. We also varied the input variables over wide plausible ranges. The sensitivity analysis shows that our findings are most sensitive to the assumptions about fixed costs, per-pass probability of success, and the time per needle pass for ROSE. This implies that institutions should obtain good estimates of these parameters to select the best sampling protocol. Some parameters may be difficult to estimate (e.g., the cost per needle pass); however, our sensitivity analysis suggests that many of the input variables have relatively little impact on the outcome.

All of the inputs are likely to vary by context. We selected four scenarios which were designed to be illustrative rather than exact. Thus, each institution would need to obtain estimates of the input parameters to determine the best sampling protocol in their particular context. Our model can help guide the collection of this information. One can use approximate estimates and use these to determine the inputs which have the greatest impact on the cost difference at their institution. They could then obtain refined estimates for the key parameters. Our sensitivity analysis suggests that the economics are driven by three to four variables.

Our model has several limitations. For example, ROSE provides qualitative benefits that are not captured by our quantitative model. For example, ROSE can lead to faster diagnosis. Also, ROSE can triage samples to appropriate ancillary testing such as immunohistochemistry, flow cytometry, molecular diagnostics and microbial culture. Thus, our model may underestimate the overall value of ROSE.

Our model has several strengths. First, our model is very general. Clinical studies have limited generality because technical inputs, operational inputs, and costs vary between institutions. For example, the probability of the success per case, *P*(*S*), depends on the number of needle passes and the per-pass probability of success, *p*. The per-pass probability of success can depend on the case-mix (anatomic location, tumor size, referral pattern), and the number of needle passes can depend on the sampling procedures or confidence of the pathologist at a particular institution. Similarly, operational factors (e.g., time per needle pass, set-up time) and costs (resource use, wages) will also vary between institutions. Our model is very flexible and can be used to predict the performance of a very wide range of sampling scenarios. Second, unlike clinical studies, our model is not subject to sampling error. Thus, we can predict small differences in performance that would be difficult to identify in clinical studies. Finally, modeling is much less costly than clinical studies. Of course, predictions from models must be verified by clinical studies; however, models can be used to guide clinical studies so that they are targeted at the most critical sources of uncertainty.

In summary, we developed a method to estimate the relative cost of ROSE and fixed sampling policies. Neither policy was universally superior in the scenarios we examined. The exact cost difference will depend on the context and will vary by institution. Our method provides a way to identify the most important factors and predict the cost difference in a particular context and can be individualized for a particular institution.

## Supporting Information

S1 Text Variable Definitions. (DOCX)

S2 Text Glossary. (DOCX)

S3 Text General Cost Model. (DOCX)

S4 Text Model Validation. (DOCX)
