# Supplementary material for: When Is Rapid On-Site Evaluation Cost-Effective for Fine-Needle Aspiration Biopsy?
Source: PLoS One. 2015 Aug 28;10(8):e0135466. doi: 10.1371/journal.pone.0135466 (PMC4552737; doi:10.1371/journal.pone.0135466)
Supplement: S1 Text — (DOCX) [file pone.0135466.s001.docx]

**S1 Appendix: Variable Definitions**

The symbol, π, is used to designate a generic sampling protocol: $\pi=F$for fixed and $\pi=R$ for ROSE. For example, $\bar{A}^{R}$ is the expected adequacy rate with ROSE ($\pi=R)$ sampling.

| **Variable** | **Variable**  **Type** | **Units** | **Explanation** |
| --- | --- | --- | --- |
| $\bar{c}_{np}$ | Input  Parameter | $/pass | Expected cost of a needle pass (e.g., adverse events) |
| $c_{fixed}$ |  | $/procedure | Fixed cost associated with FNAB procedure |
| $c_{pat}$ |  | $/hr | Wage rate of patient |
| $c_{var}^{\pi}$ |  | $/hour | Total variable cost |
| $c_{var,c}$ |  | $/hour | Variable cost of the cytologist |
| $c_{var,o}$ |  | $/hour | Variable cost for all resources other than the cytologist (e.g., technicians, radiologist, etc.) |
| $n_{F}$ |  |  | Stopping point (number of needle passes) |
| $n_{R}$ |  |  | Stopping point (number of adequate samples) for ROSE sampling. The procedure stops after the cytologist obtains $n_{R}$ adequate samples. |
| $p$ |  | % | Per-pass probability of success |
| $t_{pass}^{\pi}$ |  | Hours | Time per needle pass |
| $t_{setup}$ |  | Hours | Duration of the setup period |
| $\bar{A}^{\pi}$ | Output,  Random Variable | % | Expected adequacy rate |
| $\bar{N}_{pass}^{\pi}$ |  | Number per procedure | Expected number of needle passes per procedure |
| $\bar{N}_{proc}^{\pi}$ |  |  | Expected number of procedures required to obtain at least one adequate sample |
| $\bar{T}_{proc}^{\pi}$ |  | Hours | Expected time per procedure |
| $\bar{T}_{samp}^{\pi}$ |  | Hours | Expected duration of the sampling period |
| $\bar{TC}^{\pi}$ |  | $ per successful procedure | Expected total cost per successful procedure |
| $\bar{TC}_{proc}^{\pi}$ |  | $ per procedure | Expected total cost per procedure |
| $\bar{\Delta TC}$ |  | $ per successful procedure | Expected difference (ROSE – fixed) in cost per successful procedure |
