# Supplementary material for: When Is Rapid On-Site Evaluation Cost-Effective for Fine-Needle Aspiration Biopsy?
Source: PLoS One. 2015 Aug 28;10(8):e0135466. doi: 10.1371/journal.pone.0135466 (PMC4552737; doi:10.1371/journal.pone.0135466)
Supplement: S2 Text — (DOCX) [file pone.0135466.s002.docx]

**S2 Appendix: Glossary of Terms.**

Expected cost: A weighted average of the cost all outcomes weighted by the probability of each outcome. $\bar{c}=\sum_{i=1}^{n} c_{i}$ $p_{i}$ where $\bar{c}$ is the expected cost, $c_{i}$ and $p_{i}$ are the cost of outcome $i$, and n is the number of possible outcomes.

Input parameter: an argument for a function. For example, $x$ is a parameter of $y = f(x)$. The value of the function, or output, is specified by the value of the parameter, $x$.

Micro-costing: Micro-costing uses detailed data on resources utilized and the value of those resources.[^31^](#_ENREF_31) In micro costing, the unit of analysis is an individual procedure or patient.

One-way sensitivity analysis: Sensitivity analysis in which one parameter is varied at a time to determine the effect on the outcome. For example, consider the function, $z = x^{2} + \sqrt{y}$. A one-way sensitivity analysis for x would examine the change in z that results from varying x while holding y constant at some fixed value. One way sensitivity for y would examine the change in z due to change in y while holding x constant.

Opportunity cost: The value of a forgone alternative. For example, a lengthy procedure consumes time that could be used to generate profits from another procedure.

Perspective: The vantage point of an economic analysis. The perspective determines which costs and benefits are relevant. The payer perspective is the narrowest perspective only accounts for costs borne by the payer (e.g. an insurance company) over a limited time horizon. Costs to patients and to society (e.g. lost productivity) are not included. The government perspective accounts for all costs borne by the government over a time horizon. Typically, this will include all medical costs associated with an event over a lifetime. The societal perspective includes all costs including costs to the patient and lost productivity.

Probabilistic sensitivity analysis: An approach in which input parameters are sampled from a probability distribution to determine the probability distribution of the output. For example, consider the function, $z = x^{2} + \sqrt{y}$. Assume that $x$ is uniformly distributed between 1 and 3, and that $y$ is uniformly distributed between 5 and 8. The range of the distributions represents the uncertainty or a plausible range for the parameter. In probabilistic sensitivity analysis, values of $x$ and $y$ are randomly selected from their distributions and used to calculate z. The process is repeated thousands of times to determine the distribution of $z$, given the variation in $x$ and $y$. Unlike one-way analysis, all input parameters are varied by randomly selecting values from their distributions.

Procedure Type: We consider two generic types of FNAB procedures: simple and complex. The simple procedure is designed to model a palpation guided FNAB. Such a procedure is typically performed by a single physician (clinician or cytologist) who may be assisted by a technician. The simple procedure requires a short setup time and does not require sedation. The complex procedure is designed to model a guided FNAB procedure such as endoscopic ultra-sound guided FNA (EUS-FNA). Complex procedures require more resources (e.g., personnel, equipment, and supplies) than simple procedures and require a longer setup time.

Random Variable: A variable with an uncertain outcome. For example, the number heads that will be obtained in 5 flips of a coin, or the time to complete a FNAB procedure. The value of the outcome will vary in each trial (e.g. procedure time).

Sampling protocol: A policy is a deliberate set of principles used to guide decisions. In this paper, we consider two kinds of sampling procedures, guided by a stopping rule. In fixed sampling, the number of needle passes is determined beforehand and sampling stops when the number of needle passes is reached. In ROSE sampling, the number of needle passes is variable. Sampling stops when the cytologist observes an adequate sample.

Scenario: A scenario is a hypothetical combination of events. In this study, a scenario is a plausible combination of parameters. Our mathematical model has 10 parameters that, in theory, could take on any values. In practice, the values of the parameters are limited. For example, palpation guided FNAB procedures require few resources (personnel, equipment, supplies) and relatively short setup times. Complex FNAB procedures require more resources and have longer setup times. Each procedure type (simple or complex) can be performed using either sampling protocol (fixed or ROSE). In addition, the sampling is characterized by the per-pass adequacy rate (low or high). A scenario was defined as a specific combination of a procedure type (simple or complex), a per-pass adequacy rate (low or high), and stopping rules for ROSE and fixed sampling. .

Stopping rule: A condition used to determine when to stop sampling. The stopping rule depends on the sampling protocol. In fixed sampling (i.e. sampling without ROSE), sampling stops after a fixed number of needle passes. For example, sampling stops after three needle passes. For sampling with ROSE, sampling stops after the cytologist observes a certain number of adequate samples. Typically, sampling stops after observing one adequate sample.
