# Supplementary material for: When Is Rapid On-Site Evaluation Cost-Effective for Fine-Needle Aspiration Biopsy?
Source: PLoS One. 2015 Aug 28;10(8):e0135466. doi: 10.1371/journal.pone.0135466 (PMC4552737; doi:10.1371/journal.pone.0135466)
Supplement: S3 Text — (DOCX) [file pone.0135466.s003.docx]

**S1 Supporting material: General Model**

The costs can be divided into three categories:

1. **Hourly costs (e.g. personnel)** – we refer to these as variable costs. The resource use varies during the course of the procedure. For example, limited personnel may be required during the pre-procedure and post-procedure periods than during the procedure itself. The procedure can be divided into time periods so that each time period has constant resource use. Suppose that the procedure is divided into $k_{v}$ time periods and that $m_{i}$ resources are used during period $i$. The total hourly cost for period $i$ is obtained by summing the hourly costs of the resources used in period $i$ and multiplying by the length of period $i$:

| $c_{var,i}= t_{i}\sum_{j=1}^{m_{i}} c_{var,ij}$ | S1 |
| --- | --- |

The total variable (i.e., time-based) cost is the sum of the variable cost in each period:

| $c_{var}= \sum_{i=1}^{k_{v}} c_{var,i}= \sum_{i=1}^{k_{v}} \left( t_{i}\sum_{j=1}^{m_{i}} c_{var,ij} \right)$ | S2 |
| --- | --- |

1. **Cost incurred once per procedure (e.g. anesthesia)** – we refer to these as fixed costs. Assume that there are $k_{f}$ fixed costs. The total fixed cost is the sum of the individual fixed (i.e. procedure-related) costs:

| $c_{fixed}= \sum_{i=1}^{k_{f}} c_{fixed,i}$ | S3 |
| --- | --- |

1. **Costs incurred per needle pass (e.g. costs associated with adverse events**). Assume each needle pass is associated with $k_{n}$ costs. The cost per needle pass is the sum of these costs:

| $c_{np}= \sum_{i=1}^{k_{f}} c_{np,i}$ | S4 |
| --- | --- |

The cost is a sum because The total cost associated with needle passes is the cost per pass times the expected number of passes:

| $\bar{C}_{np}= \bar{N}_{pass} c_{np}$ | S4 |
| --- | --- |

The total cost per procedure is the sum of these costs:

| ${TC}_{proc}= c_{var}+ c_{fixed}+ \bar{C}_{np}$ | S5 |
| --- | --- |

The total cost per case is the cost per procedure times the expected number of procedures per case:

| $TC= \left( c_{var}+ c_{fixed}+ \bar{C}_{np} \right)N_{proc}=\left( c_{var}+ c_{fixed}+ \bar{C}_{np} \right)/A$ = | S6 |
| --- | --- |

In general, the costs and times will depend on the sampling procedure. We add a superscript R to designate ROSE sampling and F to designate fixed sampling. For example,

| ${TC}^{R}= \left( c_{var}^{R}+ c_{fixed}^{R}+ \bar{C}_{np}^{R} \right)/A^{R}$ | S7 |
| --- | --- |

Equations 13b is a special case of equation S7. To obtain equation equation 13b, we divide the procedure into two time periods, setup and sampling, and use equation S2 to obtain the time-based costs:

| $c_{var}^{R}= t_{setup}^{R}\left( c_{var,o}^{R}+c_{var,c}^{R} \right)+ T_{samp}^{R}\left( c_{var,o}^{R}+c_{var,c}^{R} \right)$ | S8 |
| --- | --- |

We assumed that hourly resource costs are the same for each period ($c_{var,o}^{R}+c_{var,c}^{R}$). The sampling time is given by the expected number of needle passes times the time per pass:

| $T_{samp}^{R}= N_{pass}^{R}t_{pass}^{R}=\left( \frac{n_{R}}{\left( 1-sp \right)\left( 1-p \right)+p*sn} \right)t_{pass}^{R}$ | S8 |
| --- | --- |

Then, the total variable cost is:

| $c_{var}^{R}= \left( t_{setup}^{R}+\left( \frac{n_{R}}{\left( 1-sp \right)\left( 1-p \right)+p*sn} \right)t_{pass}^{R} \right)\left( c_{var,o}^{R}+c_{var,c}^{R} \right)$ | S9 |
| --- | --- |

The total expected cost of needle passes is:

| $\bar{C}_{np}^{R}= \bar{N}_{pass} c_{np}=\left( \frac{n_{R}}{\left( 1-sp \right)\left( 1-p \right)+p*sn} \right)c_{np}$ | S10 |
| --- | --- |

We did not itemize the fixed costs, and simply estimated the aggregate fixed costs, $c_{fixed}$.

Substituting equations S9, S10, $c_{fixed}$, and equation 4 into equation S7 gives equation 13b. The derivation of equation 13a from S7 is similar.
