# Supplementary material for: When Is Rapid On-Site Evaluation Cost-Effective for Fine-Needle Aspiration Biopsy?
Source: PLoS One. 2015 Aug 28;10(8):e0135466. doi: 10.1371/journal.pone.0135466 (PMC4552737; doi:10.1371/journal.pone.0135466)
Supplement: S4 Text — (DOCX) [file pone.0135466.s004.docx]

**S2: Supporting Material: Model Validation**

Our model relies on the validity of the sampling model. The operational and cost model simply apply cost accounting principles to the sampling model.

Very few studies report the per-pass probability of success. Most studies report the average per-case probability of success along with the average number of needle passes. Once can estimate the per-pass probability of success from fixed sampling data using eqn 2:

| $\bar{A}^{F}=P\left( S \right)= P\left( X>0 \right)=1-P\left( X=0 \right)=1-{(1-p)}^{n_{F}}$ | S2.1 |
| --- | --- |

Rearranging terms, the per-pass probability of success is given by:

| $p=1-\left[ 1-P(S) \right]^{\frac{1}{n_{F}}}$ | S2.2 |
| --- | --- |

We used this relation to resolve an apparent paradox in which the ROSE was found to decrease the number of needle passes as well as to increase the per-case probability of success in EUS-FNA for solid pancreatic lesions.[1,2,3]

We are aware of two studies in which authors reported the per-case probability of success as a function of needle passes.[4,5]. We were able to show the sampling data in both of these studies showed almost perfect correspondence to a binomial sampling model.[6,7]

Thus, our sampling model has successfully predicted results in clinical studies.

To our knowledge, no studies have provided cost-effectiveness data that would allow a comparison with predictions from our operational and cost model. The operational and cost models simply apply well-accepted accounting principles to our sampling model. Thus, these aspects of the model should not be controversial.

1. Iglesias-Garcia J, Dominguez-Munoz JE, Abdulkader I, Larino-Noia J, Eugenyeva E, et al. (2011) Influence of on-site cytopathology evaluation on the diagnostic accuracy of endoscopic ultrasound-guided fine needle aspiration (EUS-FNA) of solid pancreatic masses. American Journal of Gastroenterology 106: 1705-1710.

2. Perri F (2012) EUS-guided FNA of solid pancreatic masses with or without on-site cytological evaluation: More sample adequacy with less needle passes. American Journal of Gastroenterology 107: 490.

3. Schmidt RL, Adler DG (2012) EUS-Guided Fine-Needle Aspiration of Solid Masses With or Without On-Site Cytological Evaluation: No Paradox. Am J Gastroenterol 107: 1446-1447.

4. Diacon AH, Schuurmans MM, Theron J, Brundyn K, Louw M, et al. (2007) Transbronchial needle aspirates: How many passes per target site? European Respiratory Journal 29: 112-116.

5. Suzuki R, Irisawa A, Bhutani MS, Hikichi T, Takagi T, et al. (2012) Prospective evaluation of the optimal number of 25-gauge needle passes for endoscopic ultrasound-guided fine-needle aspiration biopsy of solid pancreatic lesions in the absence of an onsite cytopathologist. Digestive Endoscopy.

6. Schmidt RL (2013) Studies on the impact of onsite evaluation must account for the per-pass success rate. Digestive Endoscopy 25: 550-550.

7. Schmidt RL, Kordy MA, Howard K, Layfield LJ, Hall BJ, et al. (2013) Risk-benefit analysis of sampling methods for fine-needle aspiration cytology: a mathematical modeling approach. Am J Clin Pathol 139: 336-344.
